# Community exercise is feasible for neuromuscular diseases and can improve aerobic capacity

**DOI:** 10.1212/WNL.0000000000007265

**Published:** 2019-04-09

**Authors:** Amanda Wallace, Aleksandra Pietrusz, Elizabeth Dewar, Magdalena Dudziec, Katherine Jones, Philip Hennis, Annette Sterr, Gianluca Baio, Pedro M. Machado, Matilde Laurá, Iwona Skorupinska, Mariola Skorupinska, Karen Butcher, Michael Trenell, Mary M. Reilly, Michael G. Hanna, Gita M. Ramdharry

**Affiliations:** From Queen Square MRC Centre for Neuromuscular Diseases, Institute of Neurology (A.W., A.P., M.D., P.M.M., M.L., I.S., M.S., M.M.R., M.G.H., G.M.R.), Institute of Sport, Exercise and Health (P.H.), and Department of Statistical Science (G.B.), University College London; National Hospital for Neurology and Neurosurgery (E.D., K.J.), University College Hospitals, NHS Foundation Trust; Faculty of Health, Social Care & Education (M.D., G.M.R.), Kingston University/St George's University of London; Department of Psychology (A.S.), University of Surrey, Guildford; Charcot Marie Tooth United Kingdom (K.B.), Registered Charity Number 1112370; and Movelab (M.T.), Newcastle University, UK.

## Abstract

**Objective:**

The aim of this phase 2 trial was to ascertain the feasibility and effect of community-based aerobic exercise training for people with 2 of the more common neuromuscular diseases: Charcot-Marie-Tooth disease type 1A (CMT) and inclusion body myositis (IBM).

**Methods:**

A randomized single-blinded crossover trial design was used to compare a 12-week aerobic training program using recombinant exercise bicycles compared to a control period. The training occurred 3 times per week in community gyms local to the participants. Support was available from trained gym staff and a research physiotherapist. The 2 disease groups were analyzed separately. The primary outcome measure was peak oxygen uptake (VO_2_ peak) during a maximal exercise test, with secondary measures of muscle strength, function, and patient-reported measures.

**Results:**

Data from 23 people with CMT and 17 people with IBM were included in the analysis. Both disease groups had high levels of participation and demonstrated improvements in VO_2_ peak, with a moderate effect size in the CMT participants (Cohen *d* = 0.53) and a strong effect size in the IBM group (Cohen *d* = 1.72). No major changes were observed in the secondary outcome measures. Qualitative interviews revealed that participants valued the support of gym instructors and the research physiotherapists in overcoming challenges to participation.

**Conclusion:**

Twelve weeks of aerobic training in community gyms was feasible, safe, and improved aerobic capacity in people with CMT and IBM.

**Classification of evidence:**

This study provides Class II evidence that for patients with CMT type 1A and IBM, an aerobic training program increases aerobic capacity.

This phase 2 feasibility trial aims to establish whether community-based aerobic training is safe, feasible, and can improve cardiopulmonary fitness in 2 more common neuromuscular diseases (NMDs): Charcot-Marie-Tooth disease type 1A (CMT) and sporadic inclusion body myositis (IBM).

Aerobic deconditioning and secondary disuse muscle atrophy are common in people with NMD and are a likely consequence of reduced general activity levels. Investigations of people with CMT found they are less active than the general population^1–3^ and are deconditioned, as measured by oxygen uptake during exercise.^4^ Similar reductions in aerobic capacity have been reported in people with idiopathic inflammatory myopathy.^5^

Only 3 underpowered, uncontrolled studies have been conducted in these 2 conditions.^6–8^ These preliminary studies indicate positive benefits of aerobic training for people with CMT and IBM but there is a need for larger trials with comparison to a control. These early studies were undertaken in hospital environments with supervision by health professionals. For lifelong behavior change, taking exercise away from the hospital and into community gym facilities may be a step to encouraging self-management, supporting the transition from exercise as a medical intervention to a leisure activity.^9^ The feasibility of moving exercise interventions into leisure facilities is untested in these conditions.

There were 3 stated aims of this study: (1) calculate the effect of training on aerobic capacity; (2) ascertain whether supported aerobic training in community leisure facilities is feasible, safe, and acceptable; (3) explore secondary physical and nonphysical effects of exercise.

## Methods

### Design

We used a single-blinded crossover design to explore the effect and feasibility of aerobic exercise training in the community. This design was used in response to observations in the field that participants are more likely to stay in a study if they receive treatment.^10^ These rare diseases are challenging to recruit, so strategies to retain participants, and reduce the effect of intersubject variation in smaller samples, were key factors in this choice of design. We have prior experience of conducting crossover exercise trials.^11^

Participants were randomized to 2 groups. Group A underwent a 12-week training period (T1), an 8-week reversal period to detrain, then a 12-week control period (C2). Group B went through the control period first (C1) and the training period second (T2). We decided to also include an 8-week period between C1 and T2 for group B to maintain blinding of assessors. People with metabolic syndrome detrain within in 1–2 months,^12^ older men detrain to baseline in 4 weeks,^13^ and people with mitochondrial disease return to baseline aerobic fitness after 8 weeks.^14^ From this evidence, 8 weeks was deemed sufficient for the detraining period.

Four assessments were undertaken by blinded assessors to measure outcomes before and after the treatment and control periods. It was not possible to blind participants in an exercise trial where aerobic capacity testing was performed using a bicycle ergometer, similar to the training intervention.

Participants from both disease groups were recruited and partook in the trial concurrently, but data were analyzed separately.

### Sample size estimate

The primary purpose of this study was to explore effect on aerobic capacity and feasibility of training, but an estimated sample size was calculated combining evidence from similar studies^7,8^ and our own experience. We estimated an intrasubject coefficient of variation of 0.2, which translates into VO_2_ measurements outside the range 17–40 mL/min/kg being extremely unlikely. Using the approach based on 2 one-sided hypothesis tests for a 2 × 2 crossover trial, a total sample size of 20 participants gave at least 80% power to detect an effect of up to 1.06 in the ratio of mean VO_2_ under the treatment and the control regimes. To account for dropouts, the target sample size was set at 30 (15 in each treatment sequence) in 2 parallel studies (one for each condition) with 30 participants each.

### Standard protocol approvals, registrations, and patient consents

This study achieved NHS NRES ethical approval (ref: 11/LO/0760) and consent for participation was obtained, recorded, and filed. This study was registered on the ISRCTN clinical trials registry (ID: 99826269)

### Recruitment strategy

Potential participants were recruited from clinics and research databases of the National Hospital for Neurology and Neurosurgery, plus national clinics of colleagues from the British Myology Society for people with IBM.

Participants were included in the study if they met the following criteria: clinical and genetic diagnosis of CMT type 1A, or a clinical diagnosis of IBM, supported by histologic confirmation as per the established Griggs criteria (only Griggs definite IBM cases were included); aged 18–80; able to walk for 30 meters with or without a walking aid or orthotic devices; ability to safely mount/dismount an exercise bike unaided or with minimal assistance; and signed informed participant consent.

Exclusion criteria were presence of other significant neurologic disorders or major comorbidities; limb surgery during the 6 months prior to screening (or planned before final assessment); failure to pass the screening assessment for exercise testing; aged over 80 or under 18 years; concurrent involvement in another intervention trial; people already participating in moderate to vigorous aerobic exercise more than 3 times per week; and women of childbearing age if they were pregnant or planning to become pregnant during the study.

### Randomization

A block randomization method was used to allocate participants to groups. Block sizes of 4 were used based on random numbers generated in MATLAB (Mathworks, Cambridge, UK). The random block sequences were stored on a password-protected spreadsheet. Allocation to the groups was input and spreadsheets held by an unblinded member of staff who were not involved in screening, recruitment, assessment, or training of the participants. This ensured allocation was concealed after enrollment and consent. Allocation was stratified according to baseline disease severity scores. For the Charcot-Marie-Tooth Neuropathy Score, categories were mild (0–10), moderate (11–20), and severe (over 20). For the IBM, the IBM Functional Rating Scale (IBMFRS) categories were mild (over 28), moderate (14–28), and severe (0–14).^15,16^

### Intervention

There are no specific guidelines for exercise training in neuromuscular diseases and therefore we adapted the aerobic training protocols recommended by the American College of Sports Medicine, considered gold standard for aerobic exercise to maintain health and fitness.^17^ We also used previous protocols used in other neuromuscular exercise studies, some of which were developed by our team*.*^14^

Participants trained in their local gyms for convenience and to increase participation. Weekly sessions with professionally qualified fitness instructors were arranged for the first month, then every other week for the second and third months. Fitness instructors received face-to-face training in the intervention protocol by the research physiotherapist, and received a training manual. The research physiotherapist visited the gym at the beginning and midpoint of the training period, and conducted a telephone review every 2 weeks. Extra calls or visits were made where either the participant or the fitness instructor identified a need for more support.

Participants exercised on a bicycle ergometer 3 times per week for 12 weeks (36 sessions) working towards a duration of 30 minutes. All had heart rate monitors to set training targets. Initially the target heart rate corresponded to 60% of VO_2_ peak. The intensity was progressively increased to 70% after 4 weeks and 80% after 8 weeks. Target heart rates were calculated using the standard formula: 220 minus the participant's age. We found in some weaker participants that they struggled to meet the heart rate training levels. In these cases, we used the BORG perceived exertion ratings to ensure they were training at a sufficiently high intensity.

Each exercise session began with a 5-minute warm-up on the bicycle ergometer and ended with a 5- to 10-minute cool-down period. Participants were encouraged to exercise on alternative days to allow time for recovery and reduce general orthopedic stress.^17^ Exercise participation and symptoms such as fatigue, pain, and mood were recorded using an exercise diary. Diary completion was monitored by the research physiotherapist during fortnightly phone calls and a midway gym visit.

For the control and detraining period, participants were asked to continue their normal, prestudy activity levels. They continued to complete the exercise diary and were encouraged to complete any additional exercise or activity they had done. Participants had a monthly telephone review during the control period where general activity, fatigue, pain, and mood were recorded.

### Primary outcome

The primary outcome measure was maximum aerobic capacity measured as peak oxygen uptake (VO_2_ peak) during a symptom-limited progressive exercise test on a semi-recombinant bicycle ergometer. Oxygen uptake was measured using indirect calorimetry (Cortex Metalyzer, Biophysik, Leipzig, Germany).

Prior to undertaking a maximal exercise test, participants underwent screening to ensure there was no evidence of cardiac dysfunction and therefore minimal risk of cardiac events during the protocol. ECG and blood pressure were monitored before, during, and after the test.

### Secondary outcomes

A battery of body structure and function, impairment, activity, and self-reported measures was used to ascertain additional effects of the aerobic training. Body structure and function measures were as follows: body mass index (BMI) and percentage body fat, measured using skin fold calipers; blood pressure and lung function (hand-held spirometry); fatigue severity using the Fatigue Severity Scale; pain, using a visual analogue scale; and isometric and isokinetic lower limb muscle function, using Cybex HUMAC dynamometer. Activity measures were maximum work rate during the exercise testing (W); 10-meter timed walk; 6-minute walk distance; and perceived walking function using the Walk-12 scale plus physical activity measured using Sensewear activity monitors for 1 week at the start of the trial and 1 week at the end of the training phase. Disease-specific measures were used to ascertain impairment and disability: CMT Examination Score (CMTES) for participants with CMT and the IBMFRS for participants with IBM. Other measures were self-efficacy for managing chronic diseases (6-item scale); barriers to activity and exercise; Short Form–36 quality of life measure; Pittsburgh Sleep Quality Scale; Epworth Sleepiness Scale; and International Physical Activity Questionnaire.

### Safety monitoring

During the training and control periods, participants recorded pain and fatigue levels using visual analogue scales. This was monitored by the research team during telephone reviews and the measurement sessions. Blood creatine kinase levels were recorded at each of the measurement sessions to monitor any change that could be indicative of muscle damage.

### Statistical analysis

The main analysis was descriptive as this phase 2 exploratory study was not powered for efficacy, and the size of effect was calculated for the primary outcome only. Means and SDs were calculated for continuous and interval data, with medians and interquartile ranges used for categorical measures. Missing data postintervention were imputed using a missing at random assumption. Where preintervention primary outcome data were missing (e.g., if participants were unable to undergo exercise testing due to raised blood pressure), the data for that individual were not included in the effect size analysis. We calculated the size of the effect of training on VO_2_ peak using both the Cohen *d* and Hedges *g* statistics, to account for any clustering within individuals. Data for participants who withdrew partway through the study were included for the time points prior to their exit.

### Qualitative analysis

Study participants were invited for a semi-structured telephone interview once they had completed the final assessment. The interviewer was not part of the main intervention team and was unknown to the participant (A.S.). Questions centered on attitudes to exercise, experience of being in the trial, and how the training intervention affected the participant. The interview was digitally recorded, transcribed, and coded using thematic analysis for emerging themes.

### Post-trial follow-up

All participants were telephoned by the research physiotherapist 3 months after the cessation of the study to see if they were continuing to exercise.

### Data availability

Any data not published within the article will be shared in an anonymized format, by request from any qualified investigator.

### Classification of evidence

This intervention study provides Class II evidence for the primary research question: What is the effect of community-based aerobic exercise training (for 12 weeks, 3 times a week at 80% of maximum heart rate) on aerobic capacity in people with CMT and IBM?

For the secondary aims, this study provides Class II evidence for the second research question: Is the intervention safe and acceptable? It also provides Class III evidence for the third research question: Are there effects on physical function, lower limb strength, fatigue, sleep, physical and quality of life?

## Results

### Recruitment

**Figure 1:**
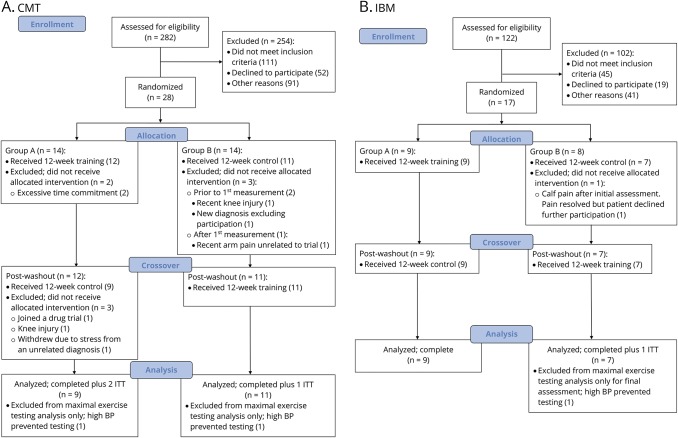
Consolidated Standards of Reporting Trials diagram Recruitment, enrollment, allocation, and retention details of participants for the (A) (CMT) and (B) (IBM) groups. BP = blood pressure; ITT = intent-to-treat.

The recruitment target for the CMT group was 30 people over a 26-month period. In total, 282 people with CMT were invited to participate and 254 were excluded, refused, or did not respond. The most common reasons for active exclusion were coexisting illness or recent limb surgery (60), already exercising over 3 days per week (32), or unable to meet time commitments (27). Thirty-one people underwent more detailed screening but 3 failed to meet the study criteria. A total of 28 people with CMT enrolled during the study period, giving a recruitment rate of 1.1 per month. Of these 28 people, 5 withdrew before starting (2 due to time commitments, 2 due to an unrelated injuries, and 1 due to a new cardiac diagnosis) and 3 partway through the study (1 joined a drug trial, 1 knee injury, 1 due to stress). In total, 20 people fully completed the study protocol, but the data for the 23 people who started the study were used for analysis using an intention-to-treat approach (figure 1).

The recruitment target for the IBM group was 30 people over a 26-month period. In total, 122 people with IBM were invited to participate and 102 were unable to commit to the trial or did not meet the study criteria on initial screening. The most common reasons were as follows: too old for the age criteria (27); did not want to participate (19); coexisting illness (18). A total of 20 people with IBM enrolled during the study period, giving a recruitment rate of 0.9 per month. Of these 20 people, 3 withdrew before being randomized (2 due to preexisting conditions, 1 was put off by the control period) and 1 withdrew part way through. In total, 16 people fully completed the study protocol, but the data for the 17 people who started the study were used for analysis using an intention-to-treat approach (figure 1).

### Participant characteristics

The CMT group was significantly younger than the IBM group, and both groups were mildly overweight, according to the mean BMI (table 1). The proportion of male to female participants was 58% male in the CMT group and 81% male in the IBM group. Both groups were moderately affected by their condition, according to the CMTES for the CMT group and IBMFRS for the IBM group (table 1).

**Table 1:**
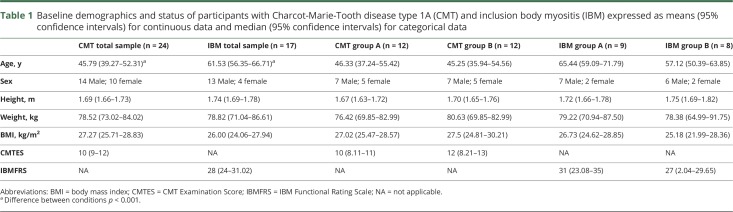
Baseline demographics and status of participants with Charcot-Marie-Tooth disease type 1A (CMT) and inclusion body myositis (IBM) expressed as means (95% confidence intervals) for continuous data and median (95% confidence intervals) for categorical data

### Participation in training intervention

The training diaries revealed that the CMT group participated in 76% of total training sessions, and the IBM group completed 91% of sessions.

### Primary outcome

**Figure 2:**
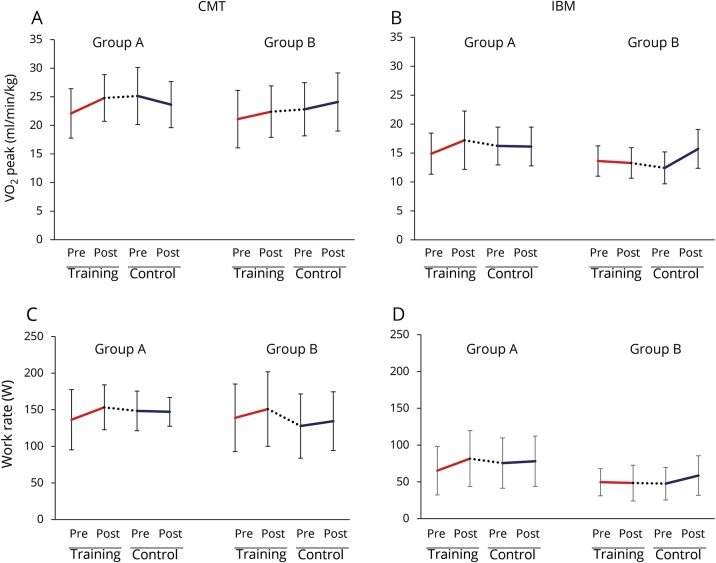
Effect of training and control interventions Line graphs of mean VO_2_ peak (mL/min/kg) for the Charcot-Marie-Tooth disease type 1A (CMT) group (A); VO_2_ peak for the inclusion body myositis (IBM) group (B); work rate (W) for the CMT group (C); work rate for the IBM group (D). Error bars are SD, solid lines represent change with the 12-week intervention/control periods, dotted lines represent change during the 8-week reversal period at crossover.

In the CMT group, the differences with training and control periods for groups A and B are detailed in table 2 and figure 2A. When the training and control data for both groups were combined, there was an 11.7% improvement in VO_2_ peak with training (pre-training 22.00 mL/min/kg, post-training 24.52 mL/min/kg) compared to a 0.1% deterioration with the control period (pre-control 23.06 mL/min/kg, post-control 22.94 mL/min/kg). The Cohen *d* and the Hedges *g* calculations for change in VO_2_ peak showed moderate treatment effect sizes.

**Table 2:**
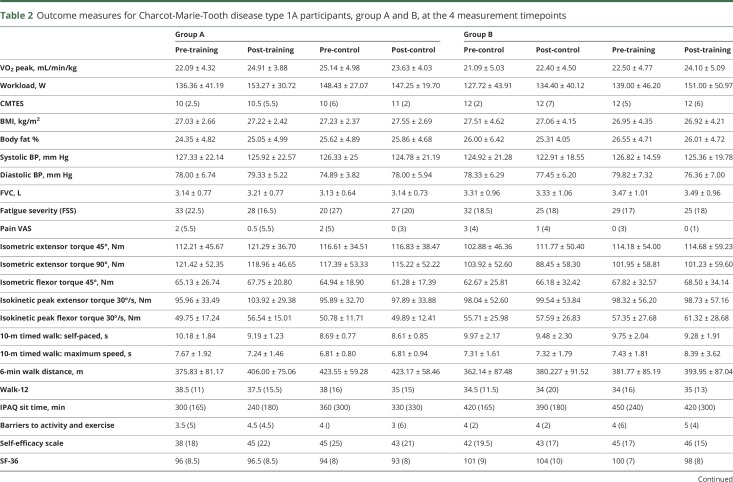

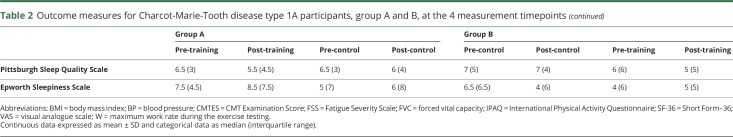
Outcome measures for Charcot-Marie-Tooth disease type 1A participants, group A and B, at the 4 measurement timepoints

In the IBM group, differences for each group are detailed in table 3 and figure 2B. Combining the training and control data for both groups showed a 17.4% improvement in VO_2_ peak with training (pre-training 14.00 mL/min/kg, post-training 16.44 mL/min/kg) compared to a 1.3% deterioration with the control period (pre-control 14.94 mL/min/kg, post-control 14.75 mL/min/kg). The effect sizes for training vs no-training were strong (table 4).

**Table 3:**
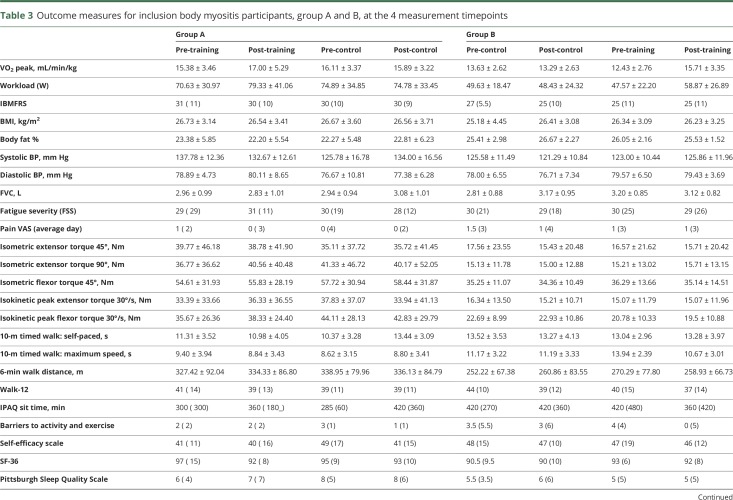

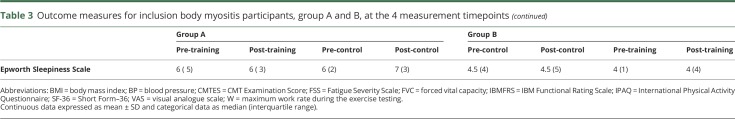
Outcome measures for inclusion body myositis participants, group A and B, at the 4 measurement timepoints

**Table 4:**
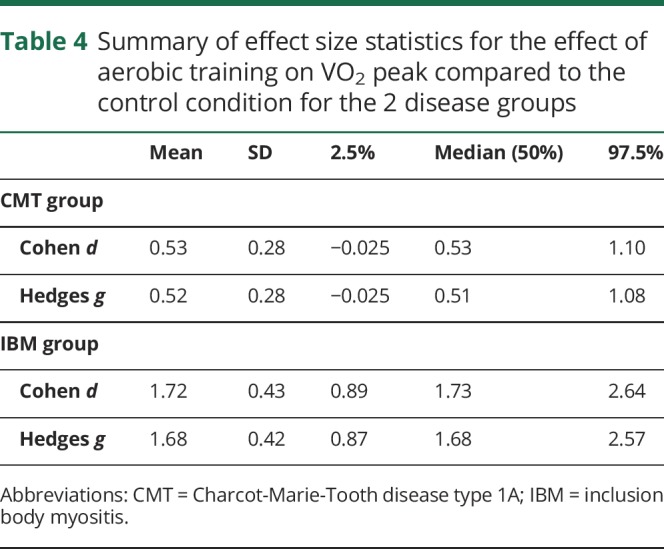
Summary of effect size statistics for the effect of aerobic training on VO_2_ peak compared to the control condition for the 2 disease groups

### Secondary outcomes

In the CMT group, when the training and control data for both groups are combined, there was a 13.1% improvement in work rate with training (pre-training 137.62 W, post-training 152.19 W) compared to a 1.5% improvement with the control period (pre-control 135.78 W, post-control 140.11 W). Differences between the 2 groups are detailed in table 2 and figure 2C. The IBM group also demonstrated changes in work rate when group data was combined. They had a 17.3% improvement with training (pre-training 59.87, post-training 70.25 W) compared to a 0.4% improvement with the control period (pre-control 63.00, post-control 63.25 W). Differences between the 2 groups are detailed in table 3 and figure 2D.

There were no major differences with training observed in the other secondary measures for the CMT and IBM participants (tables 2 and 3). A notable challenge when using the isokinetic dynamometer for very weak muscles, particularly the quadriceps muscles for the patients with IBM, was the difficulty some participants found in generating sufficient torque to trigger the motor. This may have affected the reliability of the dynamometry data.

Activity monitors administered for 7 days at the start and end of the trial showed no changes in physical activity duration over 3 metabolic equivalent tasks (CMT: pre-trial 2,490 ± 492 minutes, post-trial 2,390 ± 417; IBM: pre-trial 2,336 ± 480 minutes, post-trial 2,420 ± 424) or other physical activity variables.

### Safety monitoring

There were no increases in serum creatine kinase (CK) at group or individual level with exercise training in both conditions. There were also no changes in energy, mood, or fatigue as recorded via the visual analogue scales in the training diaries.

### Continuation of training

All participants were contacted by telephone 3 months after the trial. Contact was successfully made with 16 of the CMT participants. Eight of the 16 were still exercising, but of those who had stopped, time and work commitments were the most common reason given. Twelve participants with IBM were successfully contacted. Only 5 of the 12 were still exercising. Of the 7 who had stopped, various reasons were given, such as gym expense and access, lost confidence, time pressures, and one case of an injurious fall.

### Qualitative results

A total of 46 participants agreed to be interviewed following the trial, 32 with CMT and 14 with IBM. The experiences were common to both conditions and the following themes were identified from the data: reasons for participating in the trial; expectations; experience of participating in the trial; support; outcomes of exercise (table 5). Participants took part for altruistic and personal reasons, with the expectation that they would benefit. They expected improvements from participating in exercise and found participation in the trial acceptable. Some participants experienced physical challenges and stressed the importance of the gym instructors and research team. Overall, they described positive outcomes of the exercise intervention and expressed a desire to continue exercising, though stated that gym membership costs could be a barrier.

**Table 5:**
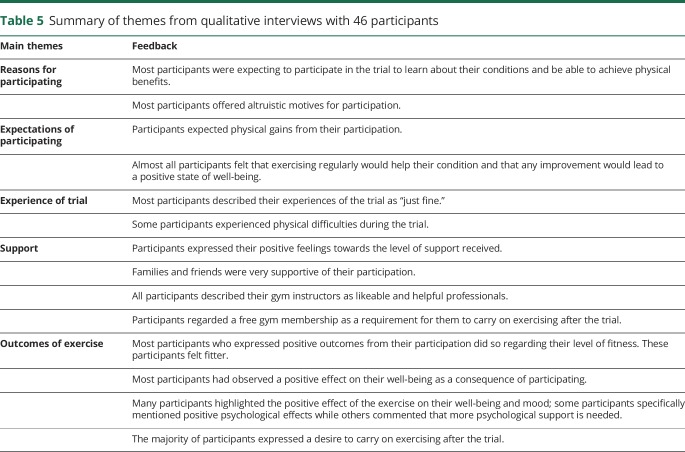
Summary of themes from qualitative interviews with 46 participants

## Discussion

It was encouraging that delivering community-based aerobic training in leisure-based exercise facilities was achievable. The excellent participation rates indicate that the approach is feasible for people with CMT and IBM; however, the low rate of continuation following the study, compared to a review of follow-up participation in other disabilities,^18^ indicates that self-motivation alone may not be sufficient for people with NMDs to continue independently. The qualitative findings indicated that support of health or exercise professionals and free or low-cost gym membership could be key factors in longer term participation in exercise. This has also been suggested from exercise trials in other neurologic conditions.^19–21^ Safety of exercise has been questioned in NMDs previously, so the lack of change in serum CK levels, pain, or fatigue is reassuring.

Both disease groups showed improvement with aerobic training, with a particularly strong effect of training in the IBM group. This group was more deconditioned at baseline and may have had greater potential for improvement, plus they showed better adherence to the study protocol. Both disease groups showed better effect sizes than training trials in other neurologic diseases.^22,23^ The effect sizes are compelling despite not meeting target recruitment for analysis in the IBM group. It is worth noting that a priori, there was an underestimation of the intrasubject coefficient of variation of 0.2, due to an assumption that VO_2_ measurements outside the range 17–40 mL/min/kg would be extremely unlikely. In the IBM group, however, 10 of the 17 participants had baseline VO_2_ measures below 17 mL/min/kg and the group mean was 14.5 mL/min/kg. This may have been due to their older age and potential greater predisposition to sedentary lifestyles. Although the CMT group presented as fitter at baseline, 3 participants were also below the 17 mL/min/kg threshold. This may have influenced the effect sizes in this study, especially with the small sample, and must be considered when estimating the sample size of a phase 3 trial. A key aim for future training trials in IBM will be to see if VO_2_ peak can be increased to greater than or equal to 17.9 mL/min/kg, the threshold for independent community living in older adults.^24^

The improvement in work rate showed similar magnitudes of change as VO_2_ peak, but none of the other secondary measures showed consistent group changes with the training or control interventions. The sample size in this study was likely to be insufficient to show change in some of the functional measures, but it may also be because the exercise intervention did not specifically train leg strength or walking. Larger samples may also be required to ascertain the effect on some of the nonmotoric factors, such as mood, fatigue, self-efficacy, and sleep. There are no studies to date that relate these factors to aerobic capacity in CMT or IBM.

There were limitations and challenges encountered during this trial that must be considered in the design of a phase 3 study. Recruitment of these rare conditions was a factor, particularly in IBM, where potential recruits tended to have more comorbidity and did not pass the safety screening for maximal exercise testing. To overcome this problem, submaximal exercise tests could be used in a phase 3 trial to reduce risk to the patient and thus increase the recruitment pool and provide a more representative sample of patient populations. Gas exchange threshold and oxygen uptake kinetic variables are ideal candidates for future studies as they are derived from submaximal exercise tests, have been successfully utilized as a markers of fitness in many clinical populations, and are responsive to exercise training.^25,26^

The crossover design was also a challenge in 2 ways. First, people were put off by the prospect of detraining if they were in the group that exercised first. This affected recruitment. In the CMT group, the VO_2_ peak did not fully return to baseline after the 8-week washout period. This will have influenced the group means and the effect sizes. If some of the recruitment challenges are addressed with a multicenter phase 3 trial and submaximal testing, a more traditional 2-arm design will be possible and would be preferable to a crossover trial.

To date, this is the largest study of aerobic exercise training in people with CMT and IBM. The protocol was conducted in community leisure facilities and demonstrated that a standard aerobic training program using a bicycle ergometer is feasible, well-tolerated, and safe for people with CMT and IBM. The prescribed program had an effect on cardiopulmonary exercise capacity and efficacy will require investigation in larger trials. Consideration must also be given to the degree of motivational and financial support required for people with NMDs to exercise on a longer-term basis as part of a self-management strategy.

## Glossary

CK: creatine kinase
CMT: Charcot-Marie-Tooth disease type 1A
CMTES: CMT Examination Score
IBM: inclusion body myositis
IBMFRS: IBM Functional Rating Scale
NMD: neuromuscular disease
W: maximum work rate during the exercise testing
